# Neoadjuvant camrelizumab and chemotherapy in patients with resectable stage IIIA squamous non-small-cell lung cancer: Clinical experience of three cases

**DOI:** 10.3389/fonc.2022.843116

**Published:** 2022-09-13

**Authors:** Xin Li, Chunqiu Xia, Minghui Liu, Jinghao Liu, Ming Dong, Honglin Zhao, Song Xu, Dan Wang, Sen Wei, Zuoqing Song, Gang Chen, Hongyu Liu, Jun Chen

**Affiliations:** ^1^ Department of Lung Cancer Surgery, Tianjin Medical University General Hospital, Tianjin, China; ^2^ Department of Pathology, Tianjin Medical University General Hospital, Tianjin, China; ^3^ Tianjin Key Laboratory of Lung Cancer Metastasis and Tumor Microenvironment, Tianjin Lung Cancer Institute, Tianjin Medical University General Hospital, Tianjin, China; ^4^ Department of Thoracic Surgery, First Affiliated Hospital, School of Medicine, Shihezi University, Shihezi, China

**Keywords:** neoadjuvant immunotherapy, non-small cell lung cancer (NSCLC), camrelizumab, major pathological response (MPR), surgery

## Abstract

Neoadjuvant immunochemotherapy has attracted much attention as a treatment for locally advanced non-small-cell lung cancer. However, there is scarce evidence of the safety and efficacy of camrelizumab as neoadjuvant in lung cancer. Here, we present three patients who were diagnosed with IIIA squamous non-small-cell lung cancer from September to December in 2020 and received two cycles of neoadjuvant camrelizumab plus nab-paclitaxel and nedaplatin, followed by surgical resection. All three patients had a reduction in the tumor size on CT image and not delayed planned surgery. We did not observe grade 3 or 4 adverse events. Two of the three patients achieved a major pathological response (MPR), including one complete tumor regression of the primary lung tumor. Multiplex fluorescent immunohistochemistry revealed that CD8+ T cells, FoxP3+ regulatory T cells, and PD-L1 expression on immune cells in the surgical specimen were much higher than in the pretreatment biopsy sample in patients with MPR. This was not observed in the patient without MPR. Camrelizumab plus chemotherapy could potentially be a neoadjuvant regimen for resectable IIIA squamous non-small-cell lung cancer, with a high MPR proportion, and did not compromise surgical procedure. Our findings should be validated in a future randomized clinical trial.

## Introduction

Neoadjuvant therapy is one of the many approaches to locally advanced non-small cell lung cancer (NSCLC) (1). However, traditional platinum-based chemotherapy either before or after resection provides only 5% higher of overall survival (OS) than surgery alone for treating patients with stage IB–IIIA NSCLC (2–4). Recently, checkpoint inhibitors targeting PD-1 and PD-L1 have revolutionized the treatment paradigm for several cancers. On the basis of previous success, several studies have focused on the utility of immune checkpoint inhibitors as neoadjuvant therapy for treating patients with surgically resectable NSCLC (5–7). Camrelizumab, a humanized monoclonal antibody against PD-1, has proved to be effective and safe in multiple tumor types including advanced NSCLC in phase 1, 2, and 3 studies (8–11). However, there has been no report regarding the efficacy and safety of the combination of camrelizumab with chemotherapy as a neoadjuvant treatment in patients with resectable lung cancer to date. Hence, we evaluated the safety and feasibility of the use of neoadjuvant camrelizumab in a small group of patients with resectable stage IIIA squamous lung cancer.

## Methods

This single-group study was developed by the author’s medical center. Three patients who were diagnosed with stage IIIA squamous lung cancer between September and December in 2020 were evaluated to undergo lobectomy surgery. Patients received the following drugs intravenously: camrelizumab (200 mg) on day 1; nab-paclitaxel (100 mg/m²) on days 1, 8, and 15; and nedaplatin (80 mg/m²) on day 1 of every 21 days for 4–6 cycles. Surgery was performed after the first two treatment cycles with an interval of 3–6 weeks (12). All the patients underwent baseline tumor assessment, including pretreatment pathological diagnosis by means of bronchoscopy or percutaneous core needle lung biopsy, contrast-enhanced CT of chest and abdomen, single photon emission computed tomography (SPECT) of bone, and magnetic resonance imaging (MRI) of brain; chest CT was repeated within 1 week before surgery. The changes in tumor size were judged according to Response Evaluation Criteria in Solid Tumors (RECIST) version 1.1. Major pathological response (MPR) was defined as the presence of 10% or less residues of cancer cells in the primary tumor surgical specimen (13).

## Results

All three patients received the two planned cycles and underwent complete tumor resection. The clinical characteristics of these patients are presented in **Table 1**. No grade 3 or 4 adverse events or treatment-related deaths occurred. Neoadjuvant camrelizumab and chemotherapy were not associated with any toxic effects, which was not previously reported. There were no treatment-related surgeries that were postponed or canceled. The intervals between the administration of the second dose of camrelizumab and surgery were 32, 39, and 41 days, respectively. Of the three patients, two received the six planned treatment cycles; one patient (patient B) was diagnosed with Alzheimer’s disease while on study and terminated the therapy after one postoperative treatment cycle. The median follow-up was 10 months (10, 11, and 12 months, respectively). None of the patients died or experienced disease recurrence during the follow-up.

**Table 1:** Characteristics of the three patients.

	Patient A	Patient B	Patient C
Pathological response	Major pathological response	Major pathological response	Incomplete pathological response
Age	68	78	68
Sex	Male	Male	Male
Smoking status	Current smoker	Current smoker	Current smoker
Histologic diagnosis	Squamous-cell carcinoma	Squamous-cell carcinoma	Squamous-cell carcinoma
Pathological stage	T2bN2M0, IIIA	T2bN2M0, IIIA	T2bN2M0, IIIA
Downstaging of nodal status	N2 to N1	N2 to N2	N2 to N2
Interval between the last dose of Camrelizumab and surgery	41	39	32
Operation time (min)	130	90	115
Bleeding (ml)	Minimal	Minimal	Minimal
Chest tube stay (days)	1	2	1
Intensive care unit stay (days)	0	0	0
Hospitalization stay following the surgery (days)	3	4	5

All three patients were operated by video thoracoscopy approach and had an R0 surgical resection. The durations of the surgery were 90, 115, and 130 min, respectively. No significant hemorrhaging occurred during surgery. The length of postoperative hospital stay was 3, 4, and 5 days, respectively, without stay in the intensive care unit (ICU). No postoperative complications, such as pneumothorax, hemoptysis, chylothorax, and pneumonia, occurred.

**Figure 1:**
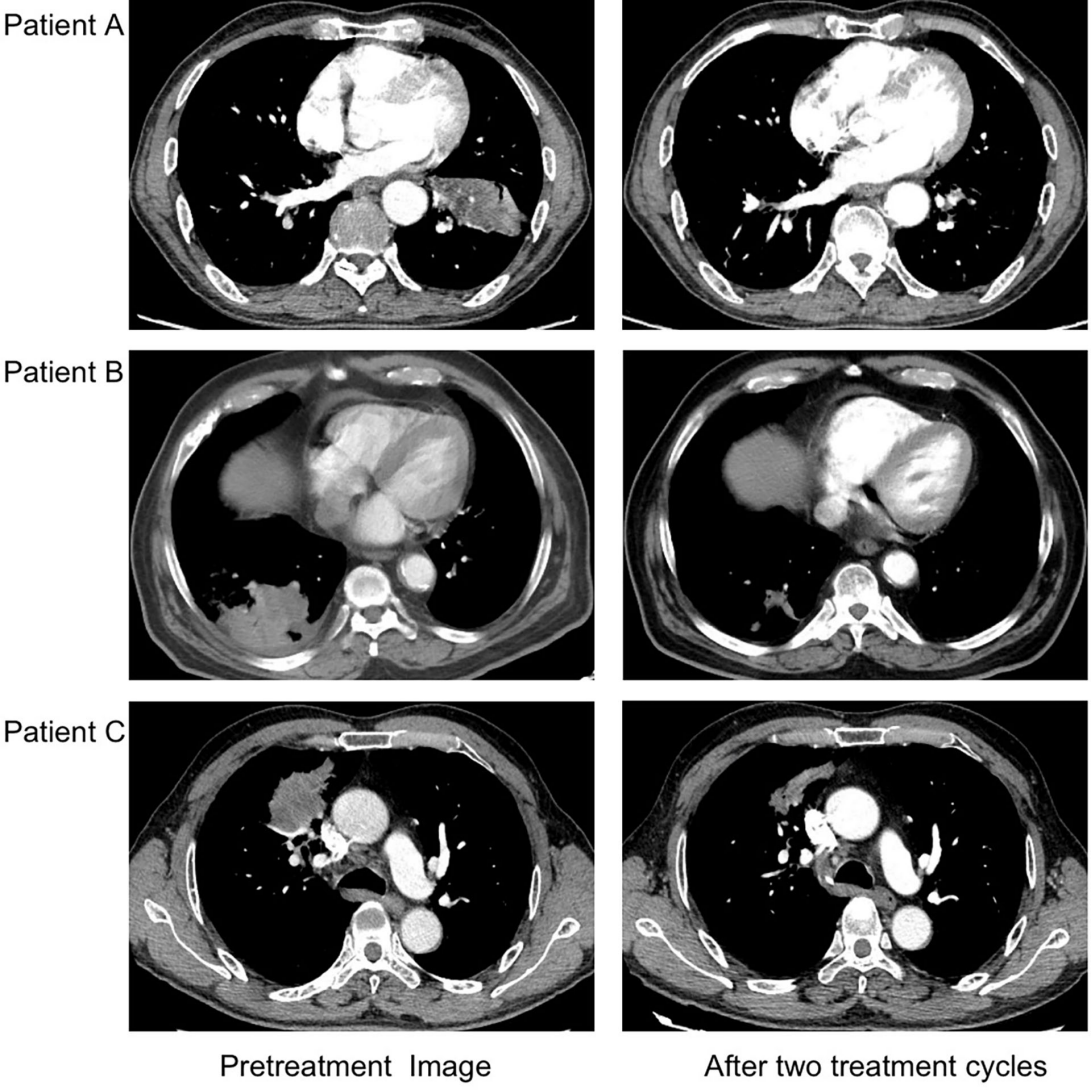
CT images of lesions before and after two cycles of neoadjuvant camrelizumab plus chemotherapy. After two doses of camrelizumab plus chemotherapy, the lesions of three patients were significantly reduced.

Two of the three patients (patient A and patient B) achieved MPR, including one complete tumor regression of the primary lung tumor but had residual lymph-node metastases (patient A). One patient (patient C) had an incomplete pathological response to the neoadjuvant treatment. Reduction in the tumor size was noted in all patients’ CT images after two cycles of treatment (**Figure 1**).

**Figure 2:**
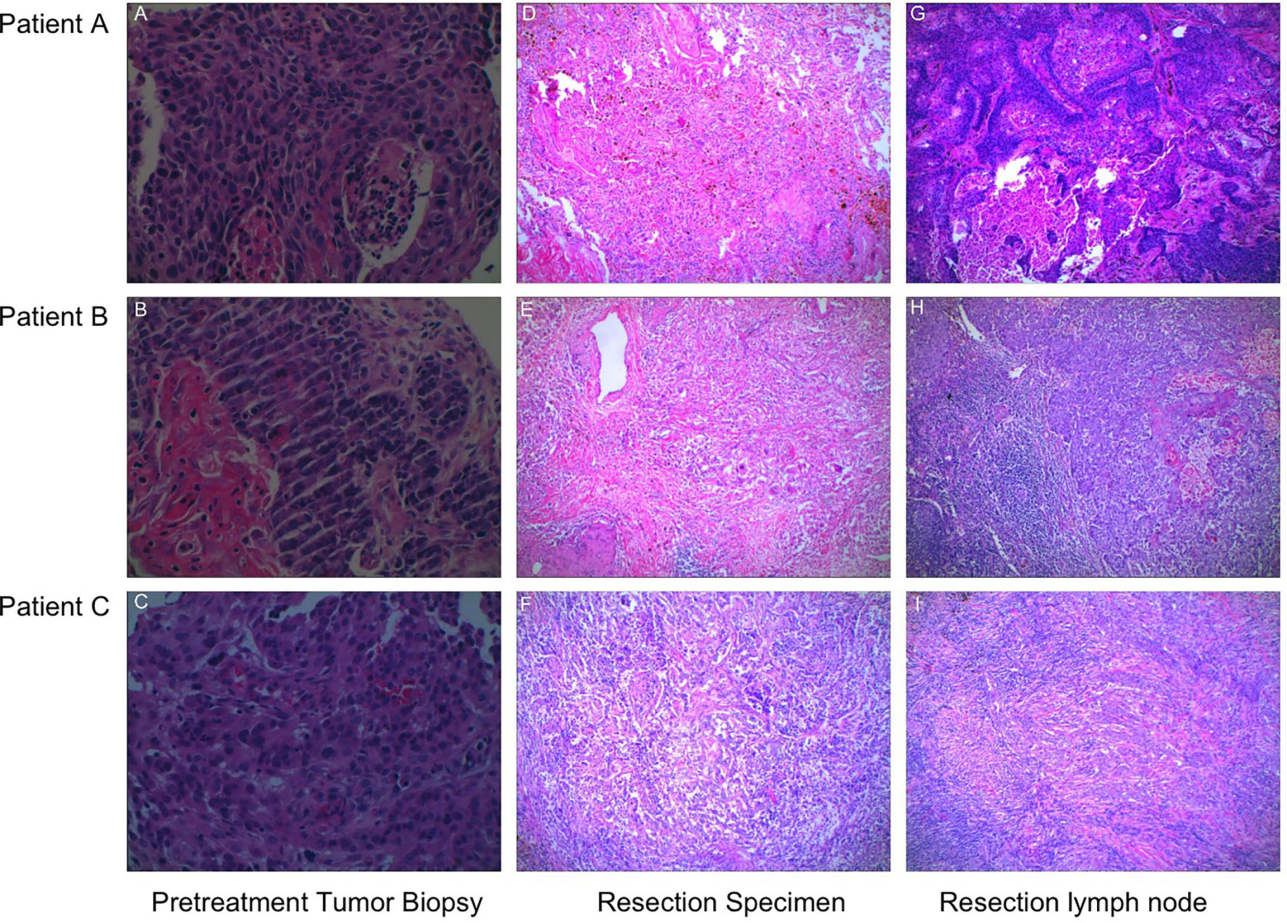
The representative sections of tumor specimens from three patients before and after the administration of camrelizumab plus chemotherapy. **(A–C)** Pretreatment tumor biopsy, HE staining (×400). **(D–E)** The resection specimens were infiltrated by lymphocytes and macrophages, and there were over 90% tumor tissue regression, HE staining (×100). **(F)** Over 50% residual tumor cells were present, and a little fibrous tissue can be observed, HE staining (×100). The presence of necrosis, fibrosis, and macrophages was observed in the metastatic lymph nodes of patients with MPR **(G, H)**, whereas this founding was not observed in the patient without MPR **(I)**, HE staining (×100).

**Figure 3:**
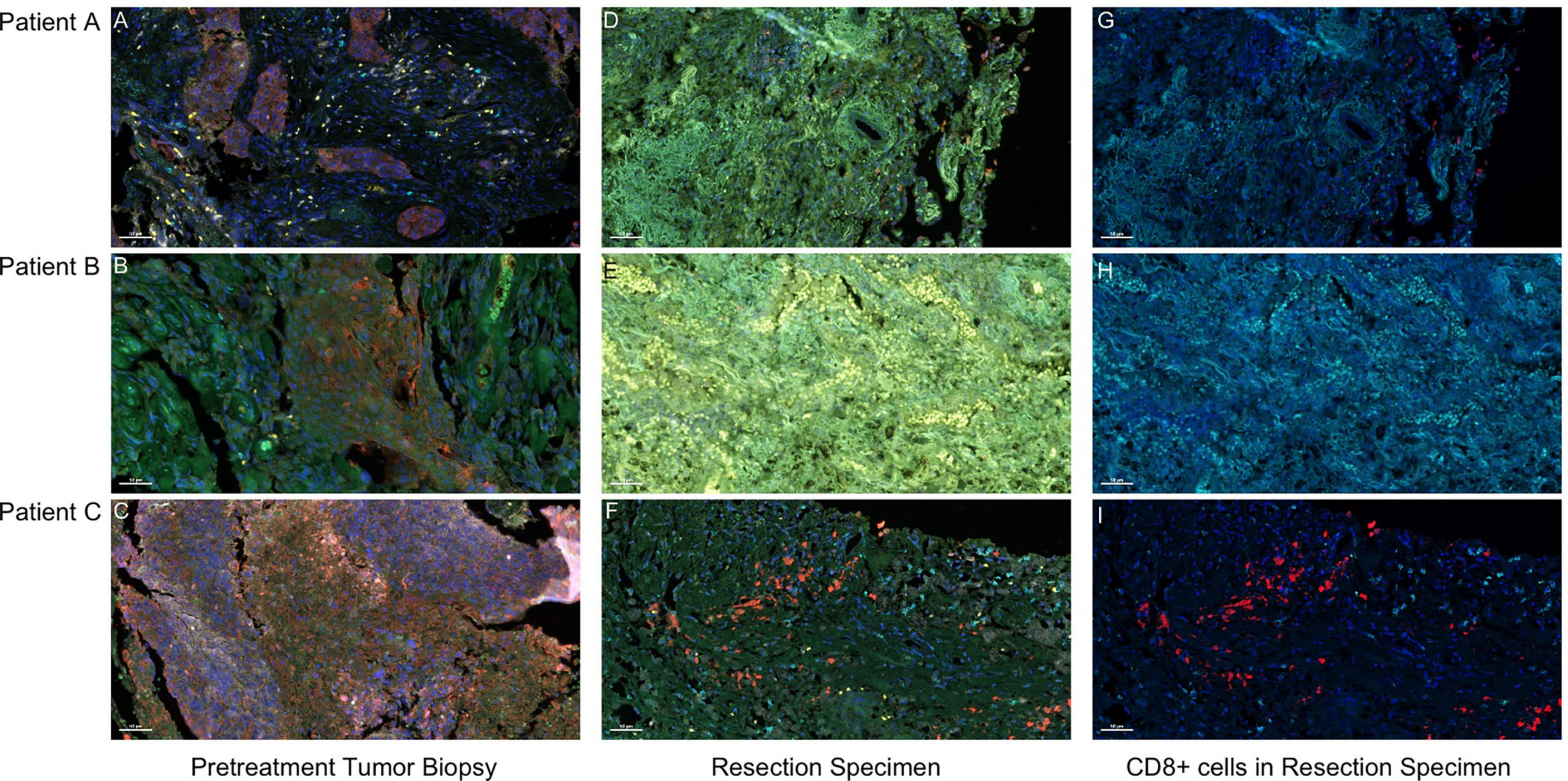
Presence of CD 68+ macrophages, CD8+ T cells, and FoxP3+ Treg cells in pretreatment and surgical specimen detected by multiplex fluorescent immunohistochemistry. Visible structures include cytokeratin-positive tumor cells (red), PD-1+ cells (orange), PD-L1+ cells (green), CD68+ macrophages (white), CD8+ T cells (cyan), and FoxP3+ regulatory T cells (yellow). **(A–C)** Pretreatment tumor biopsy tissues. After two doses of camrelizumab plus chemotherapy, the surgical specimens of patients A and B who achieved MPR contained an influx of CD8+ T cells **(G, H)**, and the presence of macrophages, Treg cells, and PD-1 and PD-L1+ immune cells were more common in the tumor area **(D, E)**. By contrast, in patient C who had no MPR, the tumor immune response was not obvious, and the amount of PD-1 and PD-L1+ immune cells was reduced after treatment **(F, I)**. Scale bar, 50 μm.

Hematoxylin–eosin staining (HE staining) revealed that residual non-viable tumor and lymph nodes of patients with MPR composed of extensive necrosis and a large amount of inflammatory cell infiltration, foamy histiocytes, and multinucleated giant cells can be seen locally. These features were not evident in areas distant from the tumor bed, which was consistent with immunological response, but this response was barely noticeable in the patient without MPR (**Figure 2**). To further explore these cases, multiplex immunofluorescence analysis which contained PD-1-positive, PD-L1-positive, CD8+ T cells, CD68+ macrophages, and FoxP3+ regulatory T cells was performed in both pretreatment and surgical specimens (**Figure 3**). In two patients with MPR, CD8+ T cell, FoxP3+ regulatory T cell, and PD-L1 expression on immune cells in the surgical specimen was much higher than in the pretreatment biopsy sample, whereas this immunoreactive intensity was faint in the patient without MPR. **Table S1** shows the changes of tumor cells and immune cells in lesions before and after neoadjuvant treatment.

## Discussion

In our study, we observed that neoadjuvant administration of two cycles of camrelizumab plus nab-paclitaxel and nedaplatin in patients with stage IIIA squamous NSCLC was not associated with additional adverse events than in previous studies (8–11). There was no delay of the planned surgery in all three patients. In addition, a radiological reduction in the tumor size was noted in all patients, which made the surgery easier to perform. Among them, two patients had MPR. The adverse events in this study were grade 1 or 2 and consistent with those of the individual drugs. The reactive cutaneous capillary endothelial proliferation that was most commonly reported related to camrelizumab was not observed in our study, which might be contributed to the combination with chemotherapy (14, 15).

Limited to traditional cytotoxic chemotherapy, advanced squamous NSCLC was associated with shorter survival than non-squamous NSCLC (16). Recently, several studies have shown that anti-PD-1 or anti-PD-L1 plus chemotherapy could improve progression-free survival and overall survival compared with chemotherapy alone in advanced squamous NSCLC (17, 18). Furthermore, MPR was more frequently observed in squamous cell carcinoma than in adenocarcinoma in neoadjuvant immunotherapy (5), which correlated with overall survival (13). The CheckMate 816 study, a phase 3 trial of neoadjuvant anti-PD-1 immunotherapy (nivolumab) plus chemotherapy versus chemotherapy alone in resectable NSCLC, reported that neoadjuvant nivolumab plus chemotherapy did not delay surgery and achieved a remarkable higher of pathologic complete response (pCR) than chemotherapy alone (24% *vs*. 2.2%). The study also showed that, compared with chemotherapy, the improvement of nivolumab plus chemotherapy on pCR was consistent in key subgroups, including disease stage (IB/II [26.2% *vs*. 4.8%]; ≥IIIA [23.0% *vs*. 0.9%]), PD-L1 tumor proportion score (<1% [16.7% *vs*. 2.6%]; ≥1% [32.6% *vs*. 2.2%]), and tumor mutational burden (low [22.4% *vs*. 1.9%]); high [30.8% *vs*. 2.7%]) (19). In terms of safety, neoadjuvant therapy with nivolumab combined with chemotherapy did not increase postoperative complications. The CheckMate 816 study reported that 11% of the patients encountered grade 3–4 surgery-relate adverse events in the nivolumab plus chemotherapy group, compared with 15% in the chemotherapy group (19). The heterogeneous ethnicities may lead to different responses to therapies. In previous studies for advanced NSCLC, the effect of camrelizumab was not inferior to nivolumab or pembrolizumab in Chinese populations (10, 11). Therefore, camrelizumab might be more suitable and economical for Chinese patients with NSCLC (10, 11). We chose nab-paclitaxel to avoid the need for steroid. Because of the concerns about serious side effects and effectiveness, patients in our study received two cycles of camrelizumab plus nab-paclitaxel and nedaplatin followed by surgical removal of the tumor, which was less than in most studies. Our results demonstrated that this shorter cycle was also very effective.

Compared to adjuvant therapy, neoadjuvant immunotherapy may improve efficacy by reducing metastasis or recurrence in early-stage NSCLC (20). PD-1 blocking enhances T-cell-mediated antitumor activity not only directly killing tumor cells but also increasing tumor antigen-specific T-cell priming (6, 21). The activated tumor-specific T cells circulate in the body to eradicate micrometastatic tumor deposits that might otherwise drive postsurgical relapse (20). Platinum-based chemotherapy could induce immunogenic tumor elimination by increasing antigen presentation, following T-cell priming (22). In our study, more infiltration of CD8+ T cells and of PD-L1 on immune cells was observed in patients with MPR after PD-1 blockade, which was consistent with an adaptive PD-L1 upregulation mechanism (23, 24). It was unexpected that patient A with complete tumor regression of the primary lung tumor had a 1.1% tumor cell PD-L1 expression in the pretreatment specimen, which was the lowest among the three patients. However, the high expression of his PD-1 on immune cells was the highest after treatment, which may contribute to the treatment response. Furthermore, the CD8+ T cells of his surgical specimen were lower than those of another patient with MPR, but his FoxP3+ regulatory T cells were also lower, which may increase immune response. By contrast, in patient C who was without MPR, the presence of PD-1 and PD-L1+ immune cells was reduced after treatment (Table S1). The limitations of our study include the small patient numbers, the absence of a randomized control group, and the short postoperative follow-up period. Larger studies are needed to correlate the pathological response resulting from neoadjuvant therapy with overall survival.

In conclusion, all three patients in this study had a radiographic response, and two of them reached a major pathological response. There were no new safety signals identified. Neoadjuvant camrelizumab plus chemotherapy could potentially be a therapeutic option for patients with stage IIIA squamous NSCLC, which requires confirmation in future prospective multicenter randomized studies.

## Data availability statement

The raw data supporting the conclusions of this article will be made available by the authors, without undue reservation.

## Ethics statement

This study was reviewed and approved by Review Board of Tianjin Medical University General Hospital. The patients/participants provided their written informed consent to participate in this study.

## Author contributions

XL, CX, ML, HL, and JC wrote the manuscript. XL, CX, ML, JL, MD, HZ, SX, SW, DW, ZS, and GC took care of the patients, collected, and analyzed the data. JC and HL supervised the research. All authors contributed to the article and approved the submitted version.

## Funding

This study was supported by grants from the National Natural Science Foundation of China (82072595, 81773207, and 61973232), Natural Science Foundation of Tianjin (18PTZWHZ00240, 19YFZCSY00040, and 19JCYBJC27000), Shihezi University Oasis Scholars Research Startup Project (LX202002), Special Support Program for the High Tech Leader and Team of Tianjin (TJTZJH-GCCCXCYTD-2-6), Tianjin Municipal Education Commission Natural Science Foundation (2019KJ202), and The Fundamental Research Funds for the Central Universities (3332018180). The funding sources had no role in study design, data collection, and analysis; the decision to publish; or the preparation of the manuscript.

## Conflict of interest

The authors declare that the research was conducted in the absence of any commercial or financial relationships that could be construed as a potential conflict of interest.

## Publisher’s note

All claims expressed in this article are solely those of the authors and do not necessarily represent those of their affiliated organizations, or those of the publisher, the editors and the reviewers. Any product that may be evaluated in this article, or claim that may be made by its manufacturer, is not guaranteed or endorsed by the publisher.
