# Supplementary material for: Neoadjuvant camrelizumab and chemotherapy in patients with resectable stage IIIA squamous non-small-cell lung cancer: Clinical experience of three cases
Source: Front Oncol. 2022 Sep 13;12:843116. doi: 10.3389/fonc.2022.843116 (PMC9514096; doi:10.3389/fonc.2022.843116)
Supplement: Supplementary file 1 [file Table_1.docx]

Table S1: Changes of tumor cells, Macrophages, T cells, and T reg cells before and after neoadjuvant treatment with camrelizumab and chemotherapy.

|  | Patient A | | Patient B | | Patient C | |
| --- | --- | --- | --- | --- | --- | --- |
| Status of neoadjuvant treatment | before | after | before | after | before | after |
| Total Cells, n (%) | 107427 (100) | 1132745 (100) | 44833 (100) | 943743 (100) | 16420 (100) | 852305 (100) |
| PanCK+ Cells, n (%) | 73761 (68.7) | 4771 (0.4) | 18127 (40.4) | 57411 (6.1) | 6458 (39.3) | 207001 (24.3) |
| CD8+ Cells, n (%) | 1451 (1.4) | 185345 (16.4) | 3717 (8.3) | 640295 (67.8) | 2018 (12.3) | 59252 (7.0) |
| CD68+ Cells, n (%) | 646 (0.6) | 7126 (0.6) | 564 (1.3) | 11381 (1.2) | 3457 (21.1) | 10360 (1.2) |
| FoxP3+ Cells, n (%) | 1475 (1.4) | 100352(8.9) | 3030 (6.8) | 304655 (32.3) | 302 (1.8) | 10595 (1.2) |
| PD-L1+ Cells, n (%) | 2702 (2.5) | 429626 (37.9) | 7628 (17.0) | 621490 (65.9) | 3721 (22.7) | 108987 (12.8) |
| PD-1+ Cells, n (%) | 761 (0.7) | 77837 (6.8) | 1906 (4.3) | 455307 (48.2) | 2631 (16.0) | 15504 (1.8) |
| PD-1+ CD8+ Cells, n (%) | 1362 (1.3) | 140156 (12.4) | 2987 (6.7) | 214578 (22.7) | 1297 (7.9) | 57567 (6.8) |
| PD-L1+ PanCK+ Cells, n (%) | 1195 (1.1) | 541 (<0.1) | 2849 (6.4) | 52701 (5.6) | 826 (5.0) | 39723 (4.7) |
| PD-L1+ CD68+ Cells, n (%) | 40 (<0.1) | 353 (<0.1) | 138 (0.3) | 6663 (0.7) | 336 (2.0) | 845 (0.1) |
| CD8+ FoxP3+ Cells, n (%) | 207 (0.2) | 76706 (6.8) | 1761 (3.9) | 299039 (31.7) | 296 (1.8) | 4444 (0.5) |
| PD-1+ CD8+ FoxP3+ Cells, n (%) | 18 (<0.1) | 26530 (2.3) | 457 (1.0) | 263725 (27.9) | 276 (1.7) | 103 (<0.1) |
| Area Analyzed (μm²) | 16411157 | 122487536 | 8284138 | 103381248 | 1970901.5 | 92000288 |
